# Resilience of bacterial quorum sensing against fluid flow

**DOI:** 10.1038/srep33115

**Published:** 2016-09-21

**Authors:** Philippe Emge, Jens Moeller, Hongchul Jang, Roberto Rusconi, Yutaka Yawata, Roman Stocker, Viola Vogel

**Affiliations:** 1 Laboratory of Applied Mechanobiology, Department of Health Sciences and Technology, ETH Zurich, 8093 Zurich, Switzerland; 2 Ralph M. Parsons Laboratory, Department of Civil and Environmental Engineering, Massachusetts Institute of Technology, 02139 Cambridge, MA, USA; 3 Department of Civil, Environmental and Geomatic Engineering, ETH Zurich, 8093 Zurich, Switzerland

## Abstract

Quorum sensing (QS) is a population-density dependent chemical process that enables bacteria to communicate based on the production, secretion and sensing of small inducer molecules. While recombinant constructs have been widely used to decipher the molecular details of QS, how those findings translate to natural QS systems has remained an open question. Here, we compare the activation of natural and synthetic *Pseudomonas aeruginosa* LasI/R QS systems in bacteria exposed to quiescent conditions and controlled flows. Quantification of QS-dependent GFP expression in suspended cultures and in surface-attached microcolonies revealed that QS onset in both systems was similar under quiescent conditions but markedly differed under flow. Moderate flow (Pe > 25) was sufficient to suppress LasI/R QS recombinantly expressed in *Escherichia coli*, whereas only high flow (Pe > 102) suppressed QS in wild-type *P*. *aeruginosa*. We suggest that this difference stems from the differential production of extracellular matrix and that the matrix confers resilience against moderate flow to QS in wild-type organisms. These results suggest that the expression of a biofilm matrix extends the environmental conditions under which QS-based cell-cell communication is effective and that findings from synthetic QS circuits cannot be directly translated to natural systems.

Cells within bacterial populations communicate in a population-density-dependent manner by quorum sensing (QS), a process based on the synthesis, release and detection of small inducer molecules^1^,^2^. A widely studied QS system is the LasI/R system in *Pseudomonas aeruginosa*, a clinically relevant gram-negative bacterium^3^. LasI synthesizes acyl-homoserine lactone (HSL) inducer molecules that are released into the surrounding fluid and – according to the classic QS model developed for quiescent environments – the local HSL concentration increases with increasing bacterial population density^4^. Above a system-specific threshold concentration, the inducer molecules bind to their cognate LasR receptors, triggering the expression of a specific set of genes, including ones involved in virulence and resistance^5^,^6^,^7^,^8^.

Natural QS systems are complex networks, which control the gene expression in bacterial populations. To reduce the system complexity and afford greater experimental control, natural QS systems are frequently isolated from the host and expressed recombinantly, often in *Escherichia coli*, using synthetic biology approaches^9^,^10^,^11^. This has yielded significant insights into the behavioral consequences and molecular details of QS. For example, the *P*. *aeruginosa* LasI/R QS system engineered into *E*. *coli* has revealed the formation of bi-directional communication across bacterial strains^12^,^13^,^14^. However, little attention has been devoted to whether the synthetic QS constructs can replicate the behavior of the original wild-type QS system under realistic environmental conditions. Here we explore this question in the context of ambient fluid flow, by comparing a wild-type and a synthetic version of the *P*. *aeruginosa* LasI/R QS system.

The traditional understanding of QS systems frequently assumes that the bacterial community – often sessile consortia called biofilms – resides in a quiescent, fixed-volume environment^15^ where the inducer molecules produced by the cells accumulate. However, the vast majority of biofilm habitats are characterized by ambient fluid flow. In nature, biofilms are prevalent in rivers, streams, intertidal regions, sinking marine particles and groundwater flow^16^,^17^,^18^. In clinical settings, biofilms form on flow-exposed catheters and implants^19^. Biofilms in the human body can experience a multitude of fluid flow regimes ranging from interstitial flow in lymphatic microvessels^20^ to laminar flow in the intestine^21^, the microvasculature^22^, and the urinary tract^23^.

It was recently shown that fluid flow can repress QS in *Staphylococcus aureus* and *Vibrio cholerae* biofilms by convective transport of the autoinducer molecules^24^, yet HSL molecules are found above threshold concentrations in samples collected from naturally occurring biofilms^18^. This implies a mechanism that counters the effect of flow on the concentration of QS signaling molecules in a biofilm, allowing naturally occurring QS systems to stay functional in flow environments. Mathematical models further revealed that flow above a biofilm can switch the QS response in biofilms suggesting that the quorum sensor acts as a flow sensor^25^ and that QS activation of bacterial colonies is substantially delayed with flow^26^.

In this study, we show that ambient fluid flow substantially delays the onset of QS in a synthetic version of the *P*. *aeruginosa* LasI/R QS system even for moderate flows, whereas in the wild-type system considerably stronger flow is needed for QS suppression. We quantified QS-dependent GFP expression in both suspended batch cultures and surface-attached bacterial microcolonies and for the latter examined quiescent conditions and conditions of flow. We found that in the absence of flow, the synthetic and natural LasI/R systems activated at similar time points. For moderate fluid flow (Peclet number >25), characteristic of many natural bacterial habitats^17^, the native *P*. *aeruginosa* QS system is activated, but not the engineered *E*. *coli* QS system. We suggest that the extracellular polymeric substance (EPS) matrix substantially reduces the wash-out of QS signaling molecules, conferring resilience to QS under flow. Lending support to this interpretation is the finding that the Psl polysaccharide – a main structural component of the EPS matrix of *P*. *aeruginosa* – is expressed before the onset of QS. This work reveals that findings from synthetic QS circuits cannot be directly translated to natural systems and that the biofilm matrix co-regulates bacterial communication under fluid flow.

## Results

### Synthetic *E*. *coli* quorum sensing reporter expresses the LasI/R QS system from *P*. *aeruginosa*

**Figure 1:**
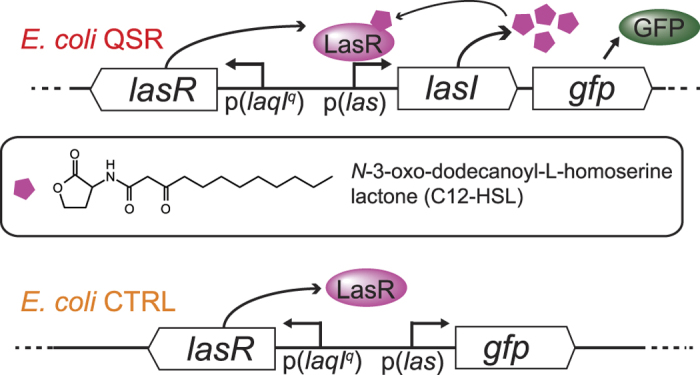
Engineered QS system in *E*. *coli*. *E*. *coli* QSR expresses *lasR* and *lasI* from a plasmid. The inducer molecule synthase LasI produces N-3-oxo-dodecanoyl-L-homoserine lactone (C12-HSL), which accumulates in the local environment with increased population density. At a C12-HSL threshold concentration, the LasR-C12-HSL complex forms and targets the expression of GFP. As negative control, a plasmid lacking *lasI* is used (*E*. *coli* CTRL).

**Figure 2:**
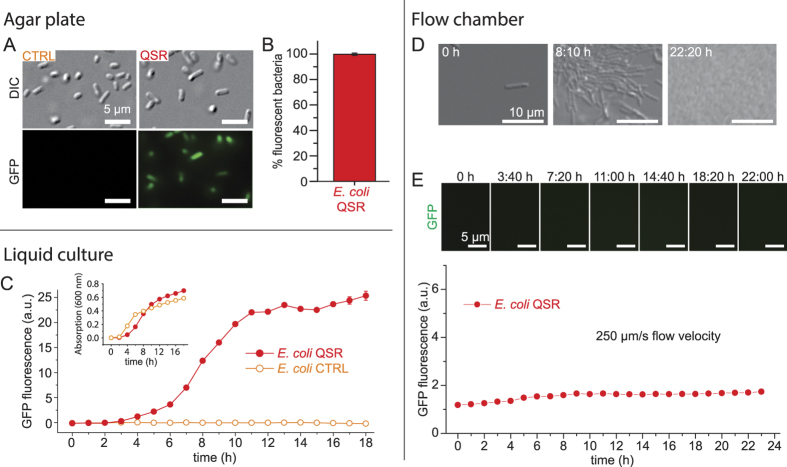
Quorum sensing in *E*. *coli* QSR is impaired by ambient fluid flow. (**A**) *E*. *coli* QSR grown on agar shows QS-dependent GFP expression. No GFP expression is detected for *E*. *coli* CTRL, lacking *lasI*. (**B**) 98% of the agar-grown *E*. *coli* QSR cells show QS-dependent GFP expression. (**C**) QS-dependent GFP expression in liquid cultures of *E*. *coli* QSR, monitored by fluorescence spectroscopy. *E*. *coli* CTRL was used as a control. Shown are the mean and standard deviation (smaller than symbol sizes) of triplicate measurements. (Inset) The growth of the two strains was very similar, as revealed by absorption measurements at 600 nm. (**D**) Growth of *E*. *coli* QSR in mannosylated glass bottom flow chamber (mean flow speed = 250 μm/s), monitored by DIC microscopy. (**E**) QS-dependent GFP expression in *E*. *coli* QSR in the same flow conditions as in D, monitored by epifluorescence microscopy. No QS-controlled GFP expression was observed. Shown are mean and standard deviation computed over *n* = 10 fields of view.

We expressed an engineered plasmid-encoded *P*. *aeruginosa* LasI/R quorum sensing reporter construct (QSR) in *E*. *coli*, where *lasI* and *gfp* are under the control of the QS-controlled promoter p(*las*) and *lasR* is constitutively expressed from the p(*laqI*^*q*^) promoter (Fig. 1). LasI synthesizes the inducer 3-oxo-C12 HSL (C12-HSL), which binds to its cognate receptor LasR. The LasR-C12-HSL complex promotes GFP expression that is used to optically monitor QS onset. The same construct but without *lasI* served as negative control (*E*. *coli* CTRL, Fig. 1). All plasmids and strains used in this study are listed in Table S1. *E*. *coli* strains were grown overnight on LB agar plates in the absence of ambient flow and colonies were analyzed by epifluorescence microscopy. 98% of *E*. *coli* QSR expressed GFP while no fluorescence was detected for *E*. *coli* CTRL cells, confirming functionality of the engineered QS constructs (Fig. 2A,B). We determined the fraction of *E*. *coli* QSR cells exhibiting QS by image thresholding and automated counting. To analyze the onset of QS in static liquid batch cultures, *E*. *coli* QSR were grown in 96-well plates without stirring, and changes in GFP fluorescence were measured with a plate reader. In fixed-volume batch cultures, the HSL inducer is predicted to accumulate with increased cell density until the threshold concentration is reached and GFP expression is triggered. We found that the GFP fluorescence of *E*. *coli* QSR increased after 3 h and plateaued at ~12 h (Fig. 2C). No fluorescence was observed for *E*. *coli* CTRL (Fig. 2C). The growth rates of *E*. *coli* QSR and CTRL were comparable, based on absorption measurements at 600 nm (Fig. 2C, inset).

### Fluid flow prevents QS in *E*. *coli* QSR

**Figure 3:**
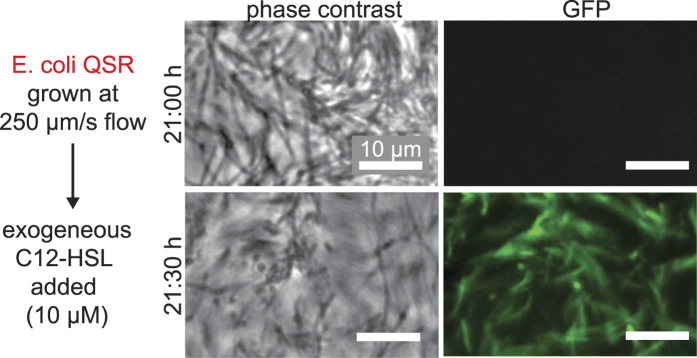
Addition of the exogenous inducer molecule C12-HSL triggers QS-dependent GFP expression in *E*. *coli* QSR also under flow. *E*. *coli* QSR grown on mannosylated glass under flow conditions (channel 1, Methods) does not exhibit QS (top GFP panel). To test for functionality, a 10-fold overdose of 10 μM C12-HSL^28^,^33^ was added to the flowing medium after 21 h of growth to ensure full activation of the LasI/R QS circuit, which resulted in strong QS-dependent GFP expression after 30 min (bottom GFP panel).

We hypothesized that ambient fluid flow constantly removes secreted HSL inducer molecules by mass transport and therefore impairs QS onset. *E*. *coli* QSR were grown under constant flow with a mean flow velocity of 250 μm/s on the mannosylated bottom glass slide of a microfluidic chamber (“channel 1”; see Methods). Coating the bottom glass slide with the mannosylated protein Ribonuclease B (RNaseB) facilitated specific FimH-mediated *E*. *coli* catch bond adhesion under elevated fluid flow^27^. Bacterial growth and QS-dependent GFP expression were monitored by differential interference contrast (DIC) and epi-fluorescence microscopy, respectively (Fig. 2D). Starting from a low initial *E*. *coli* QSR seeding density, the population density increased until bacteria were densely packed (Fig. 2D). No GFP signal was detected within the 23 h flow experiments (Fig. 2E), suggesting that the 250 μm/s flow prevented the C12-HSL inducer molecules to reach the QS threshold concentration. We confirmed that the QS reporter system is functional under flow by exogenous addition of C12-HSL to the flowing medium after 21 h, which resulted in strong GFP fluorescence in *E*. *coli* QSR after 30 min (Fig. 3).

### The synthetic *E*. *coli* QSR has the same QS threshold as the natural *P*. *aeruginosa* QSR system

**Figure 4:**
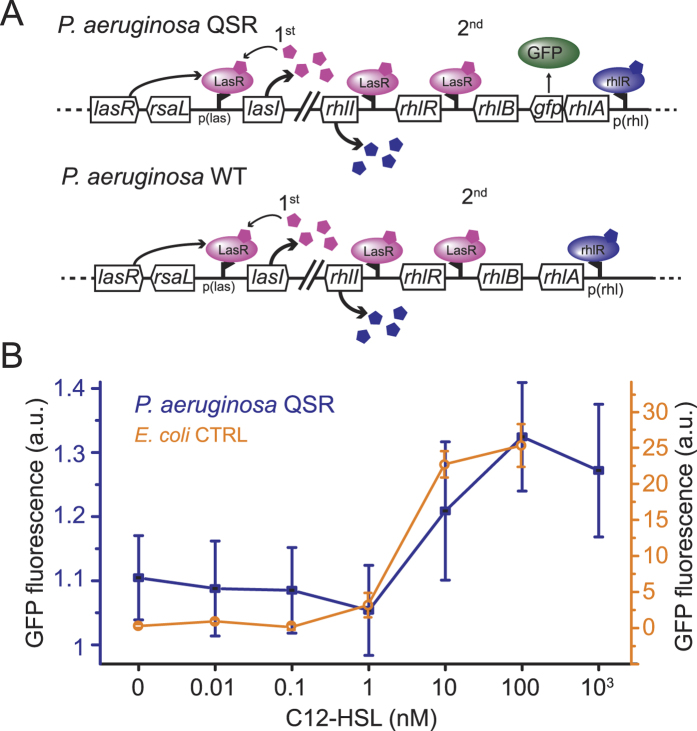
Quorum sensing has comparable C12-HSL inducer dose-response curves in P. aeruginosa QSR and *E*. *coli* CTRL. (**A**) *P*. *aeruginosa* QSR reporter construct. A *rhlA::gfp* fusion allows optical monitoring of QS onset^32^. *P*. *aeruginosa* wild type (WT), lacking the *gfp* fusion, was used as a control. (**B**) *P*. *aeruginosa* QSR and *E*. *coli* CTRL respond to similar concentrations of exogenous C12-HSL (>1 nM). C12-HSL dose response curves are obtained from GFP fluorescence of individual surface-attached *P*. *aeruginosa* QSR (n = 10 cells) and from a liquid culture of *E*. *coli* CTRL after 1 h incubation with different C12-HSL inducer concentrations. Error bars denote standard deviations.

Since QS has been reported to occur under flow conditions in Nature^19^,^28^, we asked how our findings in engineered *E*. *coli* compare to the natural system in *P*. *aeruginosa*. In the *P*. *aeruginosa* wild type strain, the expression of *rhlA* is controlled by the LasI/R and RhlI/R systems, and the activation of the RhlI/R system itself depends on LasI/R (Fig. 4A)^29^,^30^,^31^. Here, we used *P*. *aeruginosa* QSR, in which a translational *rhlA::gfp* fusion construct allows real time *in situ* monitoring of QS onset by detection of the co-expressed GFP^32^. Thus, we determined whether the synthetic QS system expressed in *E*. *coli* and the natural *P*. *aeruginosa* QS system respond to comparable inducer threshold concentrations and measured the dose response for both strains by exogenous addition of increasing inducer concentrations. For the *E*. *coli* QSR dose response, self-activation of the QS system in batch cultures was avoided by using the *E*. *coli* CTRL strain, which lacks *lasI* and therefore produces no HSL. The *E*. *coli* CTRL strain fluoresced for C12-HSL concentrations larger than 1 nM (Fig. 4B). For the *P*. *aeruginosa* QSR dose response, we performed the measurements on surface attached single bacteria under 250 μm/s fluid flow rather than in batch cultures to avoid self-activation of *rhlA:gfp*. Yet, since HSL is supplied in the flow medium, the flow itself cannot affect the local HSL concentration. GFP fluorescence was detected for C12-HSL concentrations also larger than 1 nM and maximal GFP fluorescence occurred at 100 nM (Fig. 4B). Similar C12-HSL concentrations have been reported to activate LasR-regulated genes in lacZ assays for *P*. *aeruginosa*^28^,^33^. Moreover, the increased QS response with increasing inducer concentration was similar in the two systems. This result forms the basis for the direct comparison of QS in flow across the two QS systems.

### *P*. *aeruginosa* exhibits quorum sensing in flow regimes where *E*. *coli* does not

**Figure 5:**
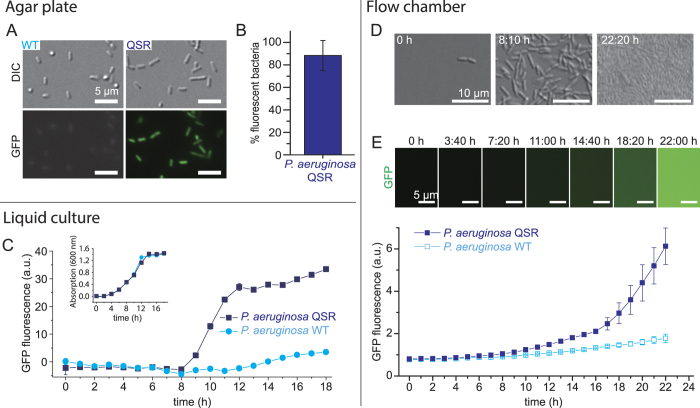
Quorum sensing in *P*. *aeruginosa* QSR is resilient to flow. (**A**) *P*. *aeruginosa* QSR grown on agar shows QS-dependent GFP expression. *P*. *aeruginosa* wild-type (WT), lacking the *gfp* fusion, has only minimal (auto)fluorescence. (**B**) 90% of the agar-grown *P*. *aeruginosa* QSR cells show QS-dependent GFP expression. (**C**) QS-dependent GFP expression in liquid cultures of *P*. *aeruginosa* QSR, monitored by fluorescence spectroscopy. *P*. *aeruginosa* WT was used as a control. Shown are the mean and standard deviation (smaller than symbol size) of triplicate measurements. (Inset) The growth of the two strains was nearly identical, as revealed by absorption measurements at 600 nm. (**D**) Growth of *P*. *aeruginosa* QSR in PLL-coated glass bottom flow chamber (mean flow speed = 250 μm/s), monitored by DIC microscopy. (**E**) QS-dependent GFP expression in *P*. *aeruginosa* QSR in the same flow conditions as in D, monitored by epifluorescence microscopy. Note the strong GFP expression despite the presence of flow. Shown are mean and standard deviation computed over *n* = 10 fields of view.

To compare the activation of the synthetic QS system expressed in *E*. *coli* with its naturally occurring counterpart, we examined the activation of the QS system in *P*. *aeruginosa*. *P*. *aeruginosa* QSR microcolonies on solid agar medium exhibited green fluorescence and 90% of cells were activated (Fig. 5A). Wild-type *P*. *aeruginosa* cells lacking the GFP reporter showed only minimal (auto)fluorescence (Fig. 5B). Liquid batch cultures of *P*. *aeruginosa* QSR started to fluoresce after 9 h of growth and plateaued at ~12 h (Fig. 5C). The signal originated from the QS-controlled GFP expression as confirmed by the lack of fluorescence from *P*. *aeruginosa* WT that does not carry the GFP reporter (Fig. 5C). Both *P*. *aeruginosa* QSR and WT showed comparable growth rates (Fig. 5C, inset), indicating that *P*. *aeruginosa* QS is activated in liquid culture when a critical population density, and thus C12-HSL concentration, is reached. To assess the flow-dependent QS behavior, *P*. *aeruginosa* QSR were grown on a poly-l-lysine (PLL)-coated glass bottom of a microfluidic chamber in the presence of a 250 μm/s flow. Starting from a low initial seeding density on the surface, the population density of *P*. *aeruginosa* QSR increased until bacteria were densely packed (Fig. 5D). Onset of QS occurred after 16 h (Fig. 5E), compared to 9 h under quiescent conditions (Fig. 5C). Autofluorescence did not contribute substantially to the GFP signal, as confirmed by comparison with *P*. *aeruginosa* WT (Fig. 5E). This result suggests that – in contrast to the results obtained in *E*. *coli* QSR (Fig. 2E) – the HSL inducer molecules in surface-attached *P*. *aeruginosa* communities accumulate sufficiently to trigger QS at 250 μm/s mean flow velocity.

### High flow rates prevent QS also in *P*. *aeruginosa*

**Figure 6:**
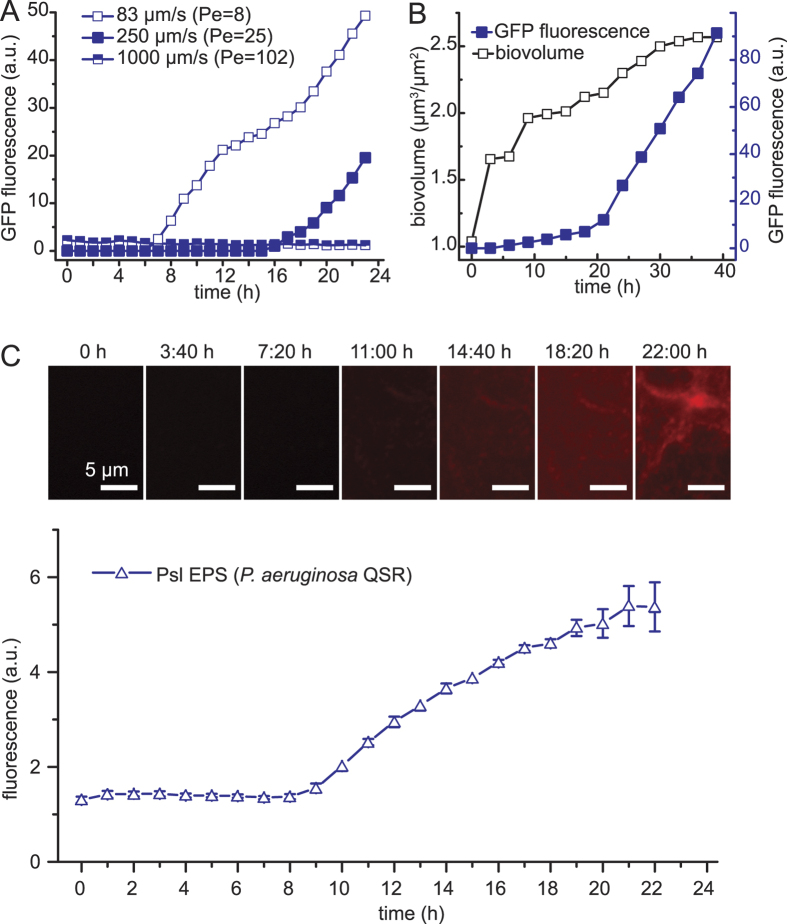
Quorum sensing under different flow velocities, biomass increase and matrix production in *P*. *aeruginosa* QSR. (**A**) QS-dependent GFP expression of *P*. *aeruginosa* QSR grown in microfluidic flows for different mean flow velocities, corresponding to different Peclet numbers Pe. (**B**) Comparison between the increase over time in the biovolume (equivalent to an average biofilm thickness) of *P*. *aeruginosa* QSR grown in a microfluidic flow (white squares; *U* = 83 μm/s), measured using confocal microscopy, and the increase over time in QS-dependent GFP expression (blue squares). (**C**) Increase over time in the production of the Psl polysaccharide by *P*. *aeruginosa* QSR grown in microfluidic flows. Psl was measured by adding to the flow medium fluorescently labeled lectin ConA that bind to Psl, and is taken as a marker for matrix production. Shown are mean values and standard deviations of *n* = 10 fields of view.

To determine the dependence of the QS response of *P. aeruginosa* on the flow magnitude, we performed experiments at different mean flow velocities. When we reduced the mean flow velocity, *U*, from 250 μm/s to 83 μm/s, the QS onset in surface-attached *P. aeruginosa* QSR was detected already at 8 h (Fig. 6A), compared to the 16 h observed at U = 250 μm/s. In contrast, at U = 1000 μm/s, QS was suppressed throughout the 23 h duration of the experiments (Fig. 6A), showing that QS is flow-dependent also in *P. aeruginosa*, where it however withstands a considerably higher flow than in *E. coli*. To obtain a quantitative measure of the importance of fluid flow in washing away inducer molecules, we compute the Peclet number of all three flows tested. The Peclet number, Pe, is a dimensionless ratio that estimates the relative importance of transport of inducer molecules by advection to their transport by diffusion. Thus, Pe = 0 in quiescent environments, when only diffusion acts, and Pe = 1 when diffusion and advection are of comparable magnitude. The Peclet number is here defined as Pe = *UH*/*D*, where *D* is the diffusion coefficient of the inducer molecules and the depth of the channel *H* was used as the characteristic length scale. For *H* = 50 μm (channel 2; Methods) and D = 4.9 × 10^−10^ m^2^ s^−1^ 4, the mean flow speeds of U = 83, 250 and 1000 μm/s correspond to Pe = 8, 25 and 102, respectively. These mean flow speeds also correspond to values of Reynolds number – i.e., the ratio between inertial forces and viscous forces in a flow, defined as Re = UH/ν, where ν is the kinematic viscosity of water – of 0.00425, 0.012 and 0.051, which confirmed the laminar nature of the flow. In all our flow experiments, the dilution of inducer molecules is dominated by convective transport by fluid flow, rather than by molecular diffusion (Peclet number >1). However, in the native *P. aeruginosa* QSR only high convective transport (Pe = 102) suppresses QS, whereas moderate convection (Pe = 25) delays it compared to weak convection (Pe = 8) but does not suppress it.

### The *P*. *aeruginosa* EPS matrix is built up before QS onset

Our results show that under flow conditions the onset of the LasI/R QS system differs between the recombinant *E*. *coli* QSR and the parental *P*. *aeruginosa* QSR. In *P*. *aeruginosa*, the biofilm matrix is known to retard the effective diffusion of small molecules^34^,^35^ and to potentially retain hydrophobic compounds^36^,^37^. This leads us to hypothesize that the matrix may be responsible for retaining the hydrophobic C12-HSL QS inducer molecules, thus reducing the inducer wash-out by fluid flow. This hypothesis implies the prediction that the onset of QS under flow occurs only after the matrix is built up. To determine the relative chronology of EPS expression and QS activation, we monitored the production of EPS matrix by *P*. *aeruginosa* QSR in real time by fluorescently labeling the Psl polysaccharide – a major structural component of the matrix^38^,^39^ – and simultaneously measured the QS-dependent GFP expression. Fluorescently tagged lectin ConA, which specifically binds α-D-mannopyranosyl residues, was added to the flow medium of growing, surface attached *P*. *aeruginosa* QSR. The staining revealed that the Psl polysaccharide formed a heterogeneous, fiber-like network (Fig. 6C), similar to that previously observed in *P*. *aeruginosa* pellicles^40^. Matrix production under high flow conditions (Pe = 25, U = 250 μm/s) started after 8 h while QS-dependent GFP expression was only detected after 16 h (Fig. 6A) showing that *P*. *aeruginosa* built up their matrix several hours prior to QS onset, in line with the hypothesis that the matrix may be implied in the resilience of QS to flow. The delayed onset of QS compared to biomass increase was also detected for reduced flow (Pe = 8, U = 83 μm/s) when we quantified the kinetics of biofilm volume increase by confocal reflection microscopy^41^ in combination with the QS-induced GFP expression by epifluorescence microscopy (Fig. 6B). The confocal measurements revealed that the mean biofilm thickness, which overall reached a value of about 2.5 μm within ~40 h, slowed down its growth after ~10 h, whereas QS only initiated after ~20 h and its intensity thereafter increased at a rate faster than the rate of biomass increase (Fig. 6B). This finding quantitatively demonstrates that the increase in the overall biofilm QS signal originated from an increase in the per-cell signal, rather than from an increase in cell numbers alone.

## Discussion

We showed that ambient fluid flow strongly impairs the onset of the LasI/R quorum sensing system when expressed recombinantly in *E*. *coli* compared to the *P*. *aeruginosa* parental strain. Our results show that under quiescent conditions, where inducer molecules are diluted only by diffusion, QS is activated in both *E*. *coli* and *P*. *aeruginosa* (Figs 2A,C and 5A,C). In contrast, when there is moderate fluid flow, QS is activated in the natural *P*. *aeruginosa* QS system (Fig. 5E), but suppressed in *E*. *coli* (Fig. 2E). This observation is important since bacterial habitats are commonly subjected to fluid flow^35^,^41^,^42^ and is in agreement with results from *S*. *aureus* and *V*. *cholerae* biofilms^24^. Our result can be rationalized in terms of the Peclet number, Pe, which reveals that under the flow conditions tested here, advection was a considerably more powerful mechanism than diffusion for transporting away inducer molecules from their secretion sites in *E*. *coli* (Fig. 6). As a result, flow was observed to suppress QS in *E*. *coli* QSR, even for moderate flow velocities (*U* = 250 μm/s, corresponding to Pe = 25). At these same flow velocities, QS was activated in *P*. *aeruginosa* QSR, as evidenced from strong QS-dependent GFP expression, and only at considerably higher flow velocities (*U* = 1000 μm/s; Pe = 102) did flow suppress QS in *P*. *aeruginosa* QSR, at least for the 23 h that the experiments lasted. Within the 3 cm-long flow chambers used here, the average autoinducer molecule concentration does not substantially vary between the chamber inlet and outlet due to flow-assisted inducer transport, as it was shown for *S*. *aureus* at the end of much longer channels (~30 cm)^24^. However, in accordance to those recent findings in *S*. *aureus*, our findings for *P*. *aeruginosa* QS systems support the idea that fluid flow is an important element of a biofilm’s physical environment that can co-regulate the onset of QS as it decreases the local QS inducer concentration by convection. In particular, these results cast doubt on any direct application where results about the effectiveness of QS antagonists or biocides obtained under quiescent conditions^43^,^44^,^45^,^46^ are translated to habitats exposed to fluid flow. Instead, we suggest that screening assays for new approaches to interfere with QS should explicitly include fluid flow as a fundamental test parameter.

Why, then, did the synthetic QS system – which matched the activation behavior and dose response of its natural counterpart under quiescent conditions – display such a different behavior from the natural QS system in the presence of fluid flow? We propose that the EPS matrix of *P*. *aeruginosa* was responsible for the retention of QS inducer molecules above the threshold concentration.

The EPS matrix is known to serve several critical functions in bacterial biofilms, including in particular a greatly enhanced resistance to antibiotics, disinfecting agents and grazing predators compared to planktonic cells^47^,^48^,^49^,^50^. Being charged and hydrophobic, the EPS matrix can considerably reduce and retard the effective diffusion of small molecules^34^,^35^ and potentially retain cationic and hydrophobic compounds^35^,^37^,^51^. Without providing a direct proof, our results point at a similar effect of the EPS matrix in reducing or retarding the loss of the hydrophobic C12-HSL inducer molecules responsible for the activation of QS. Consistent with this suggestion, we find that in the presence of fluid flow (*U* = 250 μm/s) the production of EPS (monitored in terms of Psl production) begins 8 h prior to the onset of quorum sensing (Figs 5E and 6C), indicating that the EPS matrix is in place by the time QS begins. This observation also suggests that the *psl*-operon that controls the expression of the matrix component Psl is regulated independently of QS, a finding that is in agreement with the observation that the *psl*-operon is controlled by a σ^70^-promoter and not a QS target^52^,^53^. We therefore suggest that the EPS matrix prevents the dilution of QS inducer molecules by reducing their washout by fluid flow. Interestingly, we observed the retention of the HSL molecules in relatively flat *P*. *aeruginosa* biofilms with no more than 5 layers of cells compared to much thicker biofilms reported for *S*. *aureus*^24^. Using microengineered devices, it was recently shown that spatial heterogeneity of the microenvironment, e.g. the presence of a biofilm matrix, can promote QS-based differentiation in bacterial communities and that the minimal quorum is determined by the diffusive coupling to the environment^54^. Furthermore, the diffusion of public goods through the biofilm was shown to be limited by the presence of a copious amount of EPS and by fluid flow^55^, suggesting an important role for the biofilm matrix and ambient flow in cell-cell communication and bacterial physiology in realistic environments.

There may be other, physiological differences between *P*. *aeruginosa* and *E*. *coli* that drive the difference in behavior between the natural and the synthetic QS systems under fluid flow. For example, even in the absence of differences in matrix production, resilience of QS by *P*. *aeruginosa* biofilms against flow would be observed if the presence of flow caused *P*. *aeruginosa* cells to either (i) have a remarkably higher sensitivity to inducer molecules than *E*. *coli* cells with the synthetic QS circuit, whereby *P*. *aeruginosa* cells would still exhibit QS even under the reduced inducer concentrations caused by flow; or (ii) have a remarkably higher production rate of inducer molecules, which would compensate the washout of inducer molecules by the flow. These two possibilities appear to be the main alternative explanations for our observations compared to our proposed explanation based on differential matrix production: The first alternative explanation (higher sensitivity under flow) appears unlikely based on our observations that the sensitivity of *P*. *aeruginosa* QSR under flow conditions does not exceed the sensitivity of *E*. *coli* QSR under quiescent conditions (Fig. 4; both respond above 1 nM), although we cannot entirely rule out that flow reduces *E*. *coli*’s sensitivity. The second alternative explanation (higher production under flow) can also not be ruled out entirely, but appears unlikely in view of the magnitude of the production increase required to compensate the washout. This can be seen by considering the results of a simple mathematical model for the transport of the inducer molecules in the presence and absence of fluid flow, in the same geometrical and hydrodynamic conditions as in the experiments (Methods). Model results show that, to retain the same inducer concentration at the biofilm surface in the presence of fluid flow compared to quiescent conditions, the production rate would have had to increase by 6-fold for Pe = 8 and by 11-fold for Pe = 25 (Fig. S1). Although little is known about the effect of flow on inducer production, the magnitude of this increase makes this alternative explanation less plausible.

Understanding the mechanism of retention of the inducer molecules within the biofilm will be important, as it may lead to new approaches to biofilm disruption. Retention could be physical in nature, resulting for example from a reduction in the strength of the flow in the immediate vicinity of the bacteria releasing the inducer molecules and hence a lower rate of transport away from that region. Disrupting the matrix should therefore prevent QS, yet it also leads to dispersal of the biofilm^56^ and is thus not a suitable method for assessing the role of the matrix in QS. Alternatively, retention could be of chemical nature, but chemical binding of signaling molecules to the EPS must allow for the inducer molecules to remain available to bacteria for QS to be induced, ruling out for example covalent binding. Weak hydrophobic interactions between the signaling molecules and the matrix are a possibility and changing the hydrophobicity of the matrix would then change its ability to retain inducer molecules. The resulting interference with QS and its detrimental effects, such as virulence and biofilm maturation, would have broad applications in a wide range of industrial and clinical settings.

## Methods

### Bacterial strains and media

Bacterial cultures were grown from glycerol stocks in lysogeny broth (LB) medium^57^ with appropriate antibiotics (100 μg/ml ampicillin; 50 μg/ml kanamycin; 10 μg/ml chloramphenicol) at 37 °C in a shaking incubator (Infors HT) overnight. These cultures were inoculated in LB medium with the appropriate antibiotics (as above), and grown to the desired optical density, OD_600_ (see below). An overview of all plasmids and bacterial strains used in this study is provided in the supplementary information.

### Construction of quorum sensing circuits

The quorum sensing (QS) circuits for the expression of the RhlI/R and LasI/R QS systems in *E*. *coli* MG 1655 were constructed from plasmids kindly provided by Prof. Frances Arnold and Katie Brenner^12^ as described in the supplementary information. All PCR reactions were carried out in a thermocycler (Eppendorf mastercycler) following standard protocols^58^. Primers were obtained from Microsynth AG (Balgach, Switzerland). Enzymes were purchased from New England Biolabs.

### Validation of the constructed circuits on solid agar medium

The functionality of the constructed circuits and the *P*. *aeruginosa* QS reporter was confirmed by growing *P*. *aeruginosa* QSR and *E*. *coli* QSR on LB agar medium containing the appropriate antibiotics. The cells were streaked from glycerol stocks onto LB agar petri dishes and incubated at 37 °C overnight. A single colony was picked and resuspended in 50 μl PBS. The suspension was washed once in 50 μl PBS and resuspended in 10 μl PBS. This suspension was analyzed by microscopy. The dose response was measured in batch cultures (*E*. *coli* CTRL) or in individual surface-attached bacteria (*P*. *aeruginosa* QSR). *E*. *coli* CTRL was grown in LB medium to OD_600_ = 0.2. A 200 μl aliquot of this cell suspension was added to 100 μl of LB containing a serial dilution of C12-HSL (100 nM–0.01 nM) or only LB (0 nM) in a black 96-well plate (Nunclon) with optical bottom. The plate was incubated at 37 °C for 1.5 h before fluorescence intensity was measured with a plate reader (Tecan Infinite M200). *P*. *aeruginosa* QSR was grown to OD_600_ = 0.06 and used to seed a microfluidic channel. LB medium containing first no C12-HSL and then ascending concentrations (0.01–1000 nM) of C12-HSL was applied to the surface-attached cells with a syringe pump. At each concentration, the system was incubated for 1 h with continuous flow before image acquisition.

### Microfluidic assays

All microfluidic assays were performed in polydimethylsiloxane (PDMS) based microfluidic chambers facing a glass bottom. PDMS was molded from microfabricated SU-8 master on a 4 inch silicon wafer. The PDMS replica was trimmed with a scalpel and bonded to a clean coverglass after plasma activation of both surfaces for 30 s (Harrick Plasma PDC-32G). After plasma bonding, the chamber was placed on a hotplate for 10 min at 100 °C to improve bonding quality. We used 3 cm long microchannels of two cross-sections: 1 mm width and 40 μm depth (“channel 1”) or 4 mm width and 50 μm depth (“channel 2”). A syringe pump (Harvard Apparatus PHD Ultra) was used to drive LB medium with appropriate antibiotics at flow rates of 0.6 μl/min in channel 1 (corresponding to a mean flow speed of *U* = 250 μm/s (resulting in a wall shear stress of 0.0034 pN/μm^2^)) and 1.0, 3.0, and 12.0 μl/min in channel 2 (corresponding to *U* = 83, 250, and 1000 μm/s, respectively). For EPS matrix visualization, 20 μg/ml Concanavalin A (ConA, tetramethylrhodamine conjugate (Invitrogen C860) was added to the flow medium. For *E*. *coli*, the glass bottom of the microfluidic chamber was coated with 20 μg/ml RNaseB in 0.02% bicarbonate buffer for 30 min at room temperature. The mannose residues on the RNaseB glycoprotein meditated FimH-mannose catch bond *E*. *coli* adhesion^27^. For *P*. *aeruginosa*, the glass bottom was incubated with 20 μg/ml poly-L-lysine (PLL) to facilitate electrostatic adhesion. To seed the microfluidic channels, bacterial cultures in the log phase (OD_600_ = 0.4–0.6) were washed once in PBS and resuspended in PBS to a final OD_600_ of 0.02.

### Microscopy techniques

All microscopic data were acquired by phase contrast or differential interference contrast (DIC) microscopy (Nikon TE2000-E) with a 60× oil immersion objective, or by confocal reflection microscopy (CRM) and confocal fluorescence microscopy using a 40× long-working-distance objective. CRM is a variation of reflection microscopy that employs a confocal laser scanning microscope (Zeiss 510), permitting the visualization of three-dimensional samples without fixation and fluorescent labeling^59^. Biofilm samples were illuminated with a 488 or 514 nm argon laser. Conveniently, illumination with a single laser generates both reflectance and fluorescence, which were quantified separately using 470–500 nm and 505–530 nm bandpass filters, respectively, to avoid the influence of autofluorescence. The biofilm thickness was quantified by analyzing images using COMSTAT under Matlab (The Mathworks), as described previously^60^. Epifluorescence images were acquired by fluorescein isothiocyanate (Chroma 49002) and tetramethylrhodamine (Chroma 49005) filter sets. Images were acquired with a Hamamatsu C9100-02 or an Andor iXon 885 camera.

### Numerical model for the transport of autoinducer molecules

The transport of autoinducer molecules near the biofilm surface in the presence and in the absence of flow was studied by solving numerically (Matlab, The Mathworks) the one-dimensional advection-diffusion equation for the autoinducer concentration as a function of the distance from the biofilm surface, which was approximated to have a negligible thickness compared to the height of the channel. The production of autoinducer molecules was assumed to be constant and the flow was modeled as a parabolic Poiseuille flow. The Matlab code is provided in the supplementary information.

## Additional Information

**Accession codes:** The DNA sequence of plasmid pMG401 is publicly available on GenBank (KR360752).

**How to cite this article**: Emge, P. *et al*. Resilience of bacterial quorum sensing against fluid flow. *Sci. Rep.* **6**, 33115; doi: 10.1038/srep33115 (2016).

## Supplementary Material

Supplementary Information
