# Supplementary material for: Resilience of bacterial quorum sensing against fluid flow
Source: Sci Rep. 2016 Sep 21;6:33115. doi: 10.1038/srep33115 (PMC5030672; doi:10.1038/srep33115)
Supplement: Supplementary Information [file srep33115-s1.pdf]

Resilience of bacterial quorum sensing against fluid flow

Philippe Emge[1*], Jens Moeller[1*], Hongchul Jang[2], Roberto Rusconi[2,3], Yutaka Yawata[2,3], Roman Stocker[2,3#] and Viola Vogel[1#]

Supplementary information

Plasmid or strain	Description	Designation	Source
pFNK-102	Kan^r p15A, source of *rhlI*		[10]
pFNK-502-RBSII	Kan^r p15A, source of *lasI*		[10]
pFNK-202-qsc119	Kan^r p15A containing *rhlR* and *gfp*		[10]
pFNK-503-qscrsaL	Kan^r p15A containing *lasR* and *gfp*	*E. coli* CTRL	[10]
pMG401	Kan^r p15A containing *rhlR, rhlI* and *gfp*	*E. coli* QSR	This study
PAO1	*P. aeruginosa* non-mucoid wild type	*P. aeruginosa* WT	DSMZ 19880
SMC01201	Gen^r *P. aeruginosa* with a chromosomal *rhlA::gfp* transcriptional fusion	*P. aeruginosa* QSR	Gift from G. O'Toole
MG1655	F$^-$ λ^- *rph-1 E. coli* K-12		DSMZ 18039

Table S1. Plasmids, strains and designations used in this study. DNA sequence of plasmid pMG401 is publicly available on GenBank (accession code KR360752).

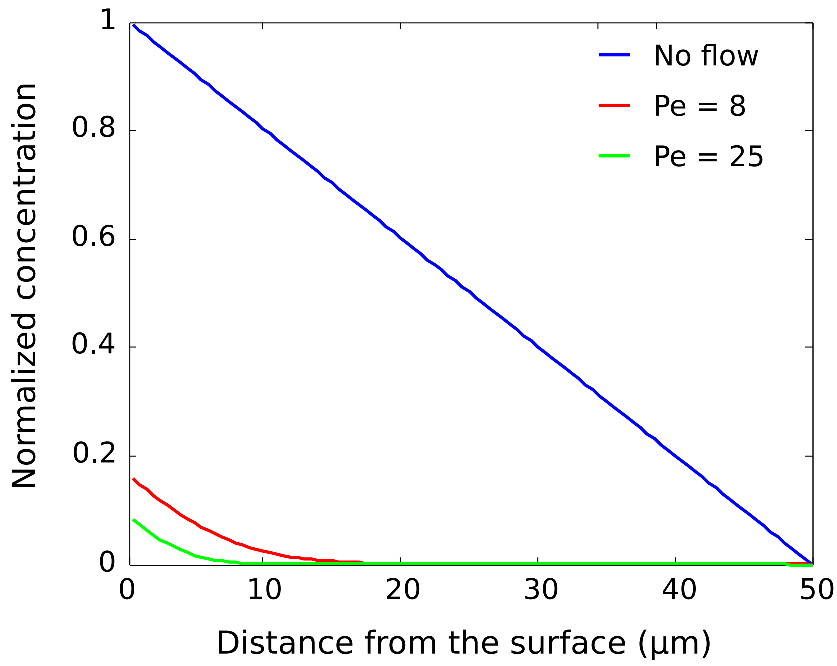

Fig. S1: Concentration of inducer above the biofilm surface, predicted with a numerical advection-diffusion model of inducer transport. In the model, the inducer is produced at a fixed rate per unit area

at the biofilm surface and its concentration is affected by molecular diffusion and advection by a flow that has a parabolic profile, in line with experiments. The boundary condition at the top of the channel is fixed to zero. The three lines denote to the steady-state results for difference flow rates, corresponding to Pe = 8 (red) and 25 (green), in addition to the no-flow case (blue). Note the strong decrease in the concentration at the biofilm surface ("Distance = 0") in the presence of flow.

Cloning of synthetic QS circuits

Starting from the cross-inducing *Pseudomonas aeruginosa* QS signaling system provided by Prof. Francis Arnold [10], we constructed a self-inducing *P. aeruginosa* QS reporter system (QSR) in *E.coli* comprising of lasR, lasI and gfp (Fig. 1). The lasI gene was PCR amplified using oligo sp_lasI-502-BamHI (5'-AAAAGGATCC AAAGAGGAGA AATTAAGCAT-3') and oligo asp_lasI-502-KpnI (5'-AAAAGGATCC AAAGAGGAGA AATTAAGCAT-3'). The PCR product was digested with BamHI and KpnI and cloned into the BamHI and KpnI sites of plasmid pFNK-503-qscrsaL, resulting in plasmid pMG401 (Table S1). The backbones were dephosphorylated by shrimp alkaline phosphatase prior to ligation. The sequence integrity of all constructs was verified by sequencing purified plasmids at Microsynth AG.

Numerical model for the transport of autoinducer molecules – Matlab code

```
function [X,U,T] = advection_diffusion_1D

m = 0;
w = 50; %width im micron
ntimes = 7;
x = linspace(0,w,101);
t = logspace(-2,4,ntimes);
u = pdepe(m,@eqn_param,@eqn_in,@eqn_bc,x,t);
U = transpose(u);
X = transpose(repmat(x,ntimes,1));
T = t;

function [c,b,s] = eqn_param(x,t,u,DuDx)
w = 50;
D = 500;
V = 2000;
c = 1;
Q = 1/6.9110e-13;
b = D*DuDx;
s = V*(1-((2*x/w-1)^2))*DuDx + Q*exp(-x*1e2);

function [pl,ql,pr,qr] = eqn_bc(xl,ul,xr,ur,t)
pl = 0;
ql = 1;
pr = ur;
qr = 0;

function value = eqn_in(x)
value = 0;
```
